# Enhanced Peroxidase-Like Activity of MoS_2_ Quantum Dots Functionalized g-C_3_N_4_ Nanosheets towards Colorimetric Detection of H_2_O_2_

**DOI:** 10.3390/nano8120976

**Published:** 2018-11-26

**Authors:** Peng Ju, Yunhong He, Min Wang, Xiuxun Han, Fenghua Jiang, Chengjun Sun, Chi Wu

**Affiliations:** 1 Institute of Marine Science and Technology, Shandong University, 72 Binhai Road, Qingdao 266237, China; jupeng@fio.org.cn; 2 Key Laboratory of Marine Bioactive Substances and Analytical Technology, Marine Ecology Center, The First Institute of Oceanography, State Oceanic Administration (SOA), 6 Xianxialing Road, Qingdao 266061, China; jiangfh@fio.org.cn; 3 Key Laboratory of Marine Chemistry Theory and Technology, Ministry of Education, Ocean University of China, 238 Songling Road, Qingdao 266100, China; yunhong_he@163.com; 4 Shandong Provincial Key Laboratory of Chemical Energy Storage and Novel Cell Technology, Liaocheng University, Liaocheng 252000, China; 13619349919@163.com; 5 Institute of Semiconductor Materials, Jiangxi University of Science and Technology, 86 Hongqi Road, Ganzhou 341000, China; xxhan@licp.cas.cn; 6 Laboratory of Marine Drugs and Bioproducts, Pilot National Laboratory for Marine Science and Technology (Qingdao), 1 Wenhai Road, Qingdao 266237, China

**Keywords:** MoS_2_, g-C_3_N_4_, peroxidase-like, colorimetric detection, H_2_O_2_

## Abstract

MoS_2_ quantum dots (QDs) functionalized g-C_3_N_4_ nanosheets (MoS_2_@CNNS) were prepared through a protonation-assisted ion exchange method, which were developed as a highly efficient biomimetic catalyst. Structural analysis revealed that uniformly-dispersed MoS_2_ QDs with controllable size and different loading amount grew in-situ on the surface of CNNS, forming close-contact MoS_2_@CNNS nanostructures and exhibiting distinct surface properties. Compared to MoS_2_ QDs and CNNS, the MoS_2_@CNNS nanocomposites exhibited a more than four times stronger peroxidase-like catalytic activity, which could catalyze the oxidation of 3,3’,5,5’-tetramethylbenzidine (TMB) in the presence of H_2_O_2_ to generate a blue oxide. Among the MoS_2_@CNNS nanocomposites, MoS_2_@CNNS(30) was verified to present the best intrinsic peroxidase-like performance, which could be attributed to the more negative potential and larger specific surface area. A simple, rapid and ultrasensitive system for colorimetric detection of H_2_O_2_ was thus successfully established based on MoS_2_@CNNS, displaying nice selectivity, reusability, and stability. The detection limit of H_2_O_2_ could reach as low as 0.02 μM. Furthermore, the kinetic and active species trapping experiments indicated the peroxidase-like catalytic mechanism of MoS_2_@CNNS. This work develops a novel, rapid, and ultrasensitive approach for visual assay of H_2_O_2_, which has a potential application prospect on clinical diagnosis and biomedical analysis.

## 1. Introduction

Over past decades, enzyme mimetics have caused extensive concern due to their favorable superiorities against harsh conditions compared to natural enzymes, such as low cost, easy preparation and storage, better stability and reusability, and nice practicability [1,2,3]. Hence, it is interesting and challenging to develop novel and effective enzyme mimetics. Recently, various enzyme mimetics have been reported and widely used in clinical diagnosis and biomedical analysis, including nanomaterials [4,5,6,7], Schiff-base complexes [8], cyclodextrin [9], hemin [10], and DNA complexes [11]. Among them, nanomaterials are becoming a novel efficient mimic peroxidase in catalyzing H_2_O_2_-mediated reaction due to their intrinsic properties like natural enzymes in size, shape and surface charge [4,5,6]. Moreover, nanomaterials exhibit nice surface properties with larger specific surface areas, more surface activation centers, and controlled catalytic potentials, which can highly promote their peroxidase-like catalytic performances [5]. Thus, the field for seeking novel nanomaterials such as peroxidase mimetics has been rapidly developed since Fe_3_O_4_ magnetic nanoparticles were first found to present the intrinsic peroxidase-like activity like that of horseradish peroxidase (HRP) in 2007 [4]. Thereafter, many kinds of nanomaterials have been exploited as peroxidase mimetics, and have exhibited good peroxidase-like properties, such as magnetic nanomaterials (CoFe_2_O_4_ [12] and FeVO_4_ [13]), carbon nanomaterials (carbon nanotubes [14], carbon dots [15], graphene oxides [16], and C_3_N_4_ [17]), noble metal nanomaterials (gold, silver and platinum) [18] and their alloys (AgVO_3_ nanobelts [19], FeSe-Pt@SiO_2_ nanospheres [20], Fe_3_O_4_-Pt nanocomposites [21], and Fe_3_O_4_-Au nanohybrids [22]), and other nanomaterials (BiOI nanoflowers [23], CeVO_4_ nanorods [24] and MoS_2_ nanoflakes [25]). Despite this progress, there is still an urgent demand to pursue novel nanomaterials with highly-efficient and stable peroxidase-like activities to overcome their inherent disadvantages, including the loss of noble metals, environmental pollution, difficulty in separation, and recyclability.

Recently, special attention has been focused on graphene-like, two-dimensional (2D) nanomaterials owing to their 2D layer structure with high energy surfaces analogous to graphene [25,26,27]. As typical 2D nanomaterials, graphitic carbon nitride (g-C_3_N_4_) possesses a stacked 2D structure and appropriate band gap (2.7 eV) owing to the *sp*^2^ hybridization of carbon and nitrogen, resulting in the formation of a stable and extended π-conjugated system [25,26,27]. And the unique graphite-like structure and tunable electronic structure of g-C_3_N_4_ lead to its large specific surface area, high thermal and chemical stability, and rapid electron transfer, accompanied by the advantage of being metal-free, abundant in natural resources, and economical, endowing g-C_3_N_4_ with extensive potential for use in new energy, sensor, and catalysis applications [25,26,27]. Currently, g-C_3_N_4_ materials have been reported as peroxidase mimetics [17], showing nice peroxidase-like activities and further extending their application areas in biotechnology. However, in view of the high recombination rate of photoinduced electron-hole pairs, the catalytic efficiency of pure g-C_3_N_4_ is greatly restricted [27]. Hence, various of methods have been performed to further improve the catalytic activity. Among them, constructing a functionalized hybrid structure using g-C_3_N_4_ as the supporter by doping with other efficient catalysts is an especially effective way, which can apparently adjust the electronic structure and accelerate electron transport [28,29]. Up to now, lots of g-C_3_N_4_-based composites have been designed and exploited to explore the synergistic enhancement effect, such as Cu/g-C_3_N_4_ [28], MnSe-g-C_3_N_4_ nanosheets [29], Fe-g-C_3_N_4_ [30], Co-g-C_3_N_4_ [31], g-C_3_N_4_/BiFeO_3_ nanocomposites [32], and so on, all of which exhibited an improved peroxidase-like activity. Therefore, in-depth investigations are indeed of great demand for designing and fabricating novel g-C_3_N_4_-based nanocomposites to further facilitate the peroxidase-like activity.

As one of the typical 2D transition metal dichalcogenides, MoS_2_ materials show excellent catalytic activity and long-term stability, which has been widely used in the fields of electronic devices, battery materials, and catalysts [33,34]. In addition, when the size decreased to less than 10 nm, MoS_2_ QDs will exhibit unique extra electrical/optical properties owing to the stronger quantum confinement and edge effects [35,36], further improving its catalytic performance. Moreover, MoS_2_ with diverse nanostructures (nanoflakes [25] and nanoparticles [37]) and several MoS_2_-based hybrids [38,39,40] have been reported as enzyme mimetics recently. Thus, on account of the matching energy band structure and nice catalytic performance, MoS_2_ QDs is becoming an ideal candidate for coupling with g-C_3_N_4_ to facilitate the peroxidase-like ability by promoting the separation and transport of electron-hole pairs. However, to the best of our knowledge, the peroxidase-like activity of MoS_2_ QDs-g-C_3_N_4_ hybrids has not been investigated until now, which deserves further and deeper exploration.

**Scheme 1:**
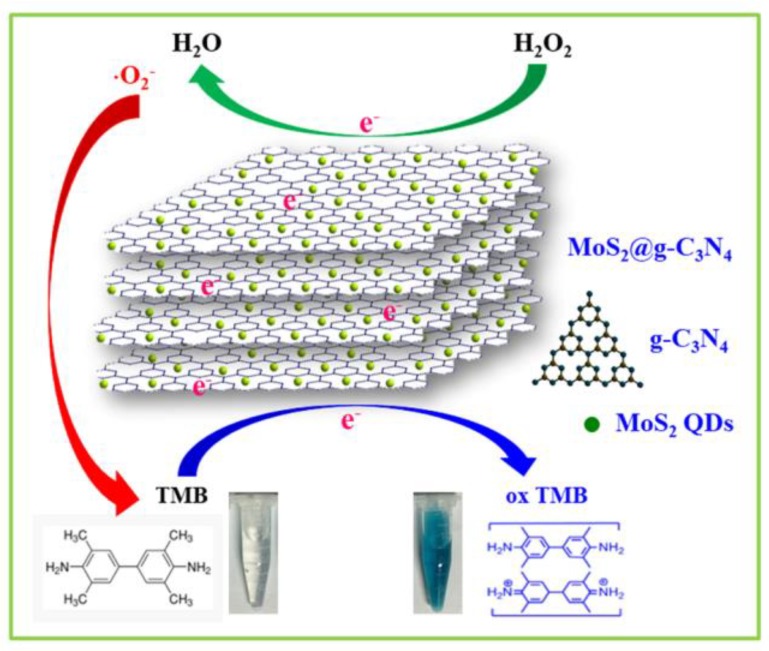
Schematic illustration of peroxidase-like catalytic reaction of MoS_2_@CNNS nanocomposites.

Herein, in view of the superiority of g-C_3_N_4_ and MoS_2_ QDs, the MoS_2_@CNNS nanocomposites with different morphologies and surface properties were successfully designed and prepared via a protonation-assisted ion exchange method, which were used as efficient artificial enzymes. With the assistance of H_2_O_2_, the MoS_2_@CNNS nanocomposites could catalyze the oxidation of the peroxidase substrate TMB to generate a blue colored reaction. Thus, a simple, rapid, ultrasensitive, selective, and stable system for the colorimetric detection of H_2_O_2_ was developed (Scheme 1). In addition, the morphology, crystal structure, surface properties, catalytic kinetics and mechanism, reusability and selectivity of the MoS_2_@CNNS nanocomposites were studied. The peroxidase-like catalytic activities of MoS_2_@CNNS nanocomposites had been greatly enhanced after the incorporation between CNNS and MoS_2_ QDs, which could be mainly attributed to the synergistic interaction, accompanied by the more negative charge and larger specific surface area. The MoS_2_@CNNS nanocomposites could have promising and broad applications in catalysis, biotechnology, and clinical diagnostics.

## 2. Materials and Methods

### 2.1. Materials and Reagents

MoS_2_ powder and 3,3’,5,5’-Tetramethylbenzidine (TMB) were purchased from Sigma-Aldrich (Shanghai, China). Melamine (C_3_H_6_N_6_), Na_2_MoO_4_∙2H_2_O, Tioacetamida (TAA, CH_3_CSNH_2_), 1-methyl-2-pyrrolidone (NMP, C_5_H_9_NO), HCl, H_2_O_2_ (3 wt %), ethanol, isopropanol alcohol (IPA), *p*-benzoquinone (BQ), FeCl_3_⋅6H_2_O, CuSO_4_⋅5H_2_O, KNO_3_, NaClO, glucose (Glu), lactose (Lac), l-valine (l-Val), glycine (Gly), and other chemicals were all of analytical grade and were purchased from Sinopharm Chemical Reagent Co., Ltd. (Shanghai, China). Lircon antiseptic liquid was obtained from Shandong Lierkang Disinfection Technology Co., Ltd. (Dezhou, China). All aqueous solutions were prepared with Milli-Q water (Millipore, Boston, MA, USA).

### 2.2. Preparation of the Catalysts

The MoS_2_@CNNS nanocomposites were prepared according to our previous report by an in-situ ion exchange method in a protonation process [36]. The bulk g-C_3_N_4_ was firstly prepared via a calcination process. Then g-C_3_N_4_ nanosheets (CNNS) were obtained by the ultrasonic exfoliation method using bulk g-C_3_N_4_ powder. After a protonation process in HCl (37%) and an ion exchange process under a hydrothermal condition, the MoS_2_@CNNS nanocomposites were finally obtained and denoted as MoS_2_@CNNS(30) (‘30’ stands for the molar ratio of Na_2_MoO_4_∙2H_2_O/CNNS was 30:1).

In addition, other MoS_2_@CNNS nanocomposites with different molar ratio were prepared as controls under the same conditions as those mentioned above, which were denoted as MoS_2_@CNNS(15) and MoS_2_@CNNS(45), respectively. Moreover, pure MoS_2_ QDs were prepared as control via an ultrasonic exfoliation method [41]. In brief, 100 mg of MoS_2_ powder was added into 10 mL of NMP and sonicated for 3.5 h. Then, the dispersion was kept stable overnight and centrifuged at 5500 r/min for 90 min. Finally, the MoS_2_ QDs were obtained by collecting the top part of the dispersion.

### 2.3. Characterization

The crystal phase and structure of the as-prepared samples were analyzed by powder X-ray diffraction (XRD) measurements on a Germany Bruker D8 Advanced powder diffractometer using Cu *K_α_* radiation (*λ* = 0.15406 nm). The morphology and microstructure of the as-prepared samples were observed by transmission electron microscopy (TEM), high resolution transmission electron microscopy (HRTEM), and selected area electron diffraction (SAED) (JEOL JEM-2100, Tokyo, Japan). Contents of S and Mo in MoS_2_@CNNS nanocomposites were measured via an inductively coupled plasma emission spectrometer (ICP-AES, Varian725-ES, Palo Alto, CA, USA). The specific surface areas were determined by an automatic nitrogen adsorption specific surface and pore size distribution analyzer (NOVA 4000e, Quantachrome Instruments, Boynton Beach, FL, USA) at 77 K after a pretreatment at 473 K for 2 h. The zeta potential tests were determined on a zeta potential measuring instrument (Zetasizer Nano-ZS, Malvern Instruments Ltd., Malvern, UK), which was examined six times (each time being the average of 100 runs) at pH 4.0, and the mean values and standard deviations were calculated automatically based on Smoluchowski’s equation.

### 2.4. Peroxidase-Like Activities and Steady-State Kinetic Assay

The catalytic oxidation experiments (a peroxidase substrate TMB with H_2_O_2_) were carried out at room temperature to comparably investigate the peroxidase-like activities of the as-prepared catalysts, including MoS_2_ QDs, CNNS and MoS_2_@CNNS nanocomposites. Typically, the tests were performed by adding 200 μL of 600 μg/mL catalysts into the reaction systems containing 500 μL of 50.0 mM phosphate buffer solution (PBS, pH = 4.0), 100 μL of 8.0 mM TMB, and 200 μL of 10.0 mM H_2_O_2_. Then, the reaction systems were monitored in a time-scan mode at 652 nm by an UV-visible spectrophotometer (Shimadzu UV-2500, Kyoto, Japan) right after all of the components were added and mixed. The effect of MoS_2_@CNNS(30) concentration (0–200 μg/mL), H_2_O_2_ concentration (0–5.0 mM), pH (2.0–9.0), and temperature (10–50 °C) on the peroxidase-like activity of MoS_2_@CNNS(30) were also tested by the same procedures mentioned above to probe the optimal reaction conditions (Supplementary Information Figure S2 and Figure S3).

The steady-state kinetic tests were performed in a time course mode at 652 nm by an UV-visible spectrophotometer under the optimal experimental conditions (25.0 mM PBS, pH = 4.0, 25 °C, 120 μg/mL MoS_2_@CNNS(30)) by varying the concentration of TMB at a fixed concentration of H_2_O_2_ or vice versa [4,42]. The Michaelis-Menten constant was measured using the Lineweaver-Burk double reciprocal plot according to the equation 1/*v* = (*K_m_*/*V_max_*) × (1/[*S*]) + 1/*V_max_* [4,42], where *v* is the initial reaction velocity, *K_m_* is the Michaelis-Menten constant, *V*_max_ is the maximum reaction velocity, and [*S*] is the concentration of substrate.

### 2.5. Analysis of Active Species

To explore the roles of active species played in the catalytic reaction, the active species trapping experiments were carried out. In brief, scavengers (10.0 mM IPA, a scavenger of ⋅OH; 10.0 mM BQ, a scavenger of ⋅O_2_^−^) were added into the reaction systems respectively to remove the active species under the optimal experimental conditions [23,24,43]. Then, the absorbance and color change of the reaction system was recorded at 652 nm by an UV-visible spectrophotometer.

### 2.6. H_2_O_2_ Detection

A colorimetric detection system of H_2_O_2_ was set up based on the nice peroxidase-like performances of MoS_2_@CNNS(30). The reaction system contained 100 μL of 8.0 mM TMB, 200 μL of 600 μg/mL MoS_2_@CNNS(30), 200 μL of H_2_O_2_ with different concentrations (0–0.1 mM), and 500 μL of 50.0 mM PBS (pH = 4.0). Then, the absorbance change of the mixture was recorded on time-mode at 652 nm with an UV-visible spectrophotometer to obtain a standard curve.

In addition, the specificity and selectivity of MoS_2_@CNNS(30)-based detection system were evaluated by adding some potential interfering substances into the reaction system under the same experimental conditions mentioned above, such as metal and non-metal ions (20.0 mM of Fe^3+^, Cu^2+^, NO_3_^−^, and ClO^−^) and common organic compounds (20.0 mM of glucose (Glu), lactose (Lac), L-valine (L-Val), and glycine (Gly)). The practical detection assay of H_2_O_2_ based on MoS_2_@CNNS(30) was performed by adding commercial antiseptic solution (containing about 0.79 M H_2_O_2_) into the reaction system instead of standard H_2_O_2_ with the other operations fixed. The antiseptic liquid was diluted 1000 times before the test.

### 2.7. Stability and Reusability of the Catalysts

The stability and reusability of MoS_2_@CNNS(30) were studied by recycling the H_2_O_2_ detection assays 10 times under optimal experimental conditions. The reaction system was monitored at 652 nm by a UV-visible spectrophotometer for 10 min. After each cycle, the MoS_2_@CNNS(30) samples were collected by centrifugation, washed with Milli-Q water and alcohol, dried at 60 °C for 30 min, and then reused in the next cycle. The crystal structure and morphology of MoS_2_@CNNS(30) samples after 10 cycles were characterized by XRD and TEM as described above.

## 3. Results

### 3.1. Characterization of the Catalysts

**Figure 1:**
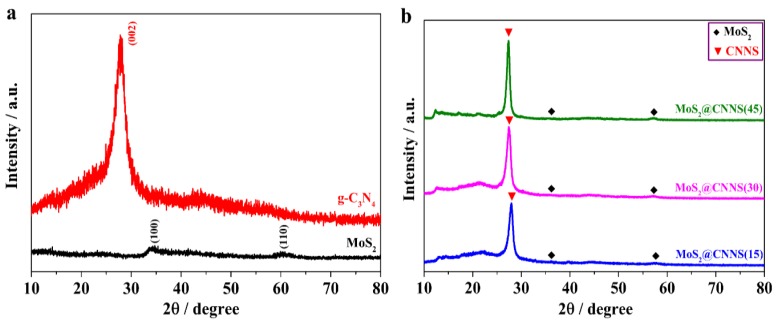
XRD patterns of MoS_2_ QDs and g-C_3_N_4_ (**a**) and MoS_2_@CNNS nanocomposites (**b**).

The crystal phase, structure, and crystallinity of the as-prepared samples were determined by XRD. It can be seen in Figure 1a that two tiny diffraction peaks can be barely seen in the XRD pattern of MoS_2_ QDs, which can be attributed to the (100) and (110) lattice planes of MoS_2_, respectively. However, the two diffraction peaks were extremely weak, which implied that the MoS_2_ QDs had poor crystallinity [36]. In addition, the CNNS samples show a strong peak at 27.29°, which can be attributed to the characteristic of the stacking peak of π-conjugated layers and indexed for the interlayer reflection of a graphite-like structure as the (002) peak [29,36,44], consistent with the literature value (JCPDS No. 87-1526). Moreover, the absence of a (100) peak and the presence of a sharp (002) peak further confirmed the layered structures of CNNS samples and the good crystallinity of CNNS. As for the MoS_2_@CNNS composites (Figure 1b), all the characteristic diffraction peaks can be well indexed to the graphite-like structure of CNNS (JCPDS No. 87-1526) and hexagonal phase MoS_2_ (JCPDS Card No. 37-1492), indicating that MoS_2_ QDs were successfully formed on the surface of CNNS through the aid of ion-exchange process. Furthermore, with increasing the loading amount of MoS_2_ QDs, the relative intensity of corresponding (100) and (110) diffraction peaks of MoS_2_ strengthened gradually, while the characteristic peaks of MoS_2_ were still relatively weak and broad in the composites owing to the size effect of quantum dots. There were no significant shifts of the characteristic diffraction peaks occurring in the MoS_2_@CNNS composites, implying that MoS_2_ QDs existed as a separate phase rather than being incorporated into the lattice of CNNS [36]. No impurity peaks were observed in the obtained samples, indicating the pure phase of the samples. Hence, these results indicated that the MoS_2_@CNNS nanocomposites were successfully obtained through the synergistic effect of anion-exchange and hydrothermal process.

**Figure 2:**
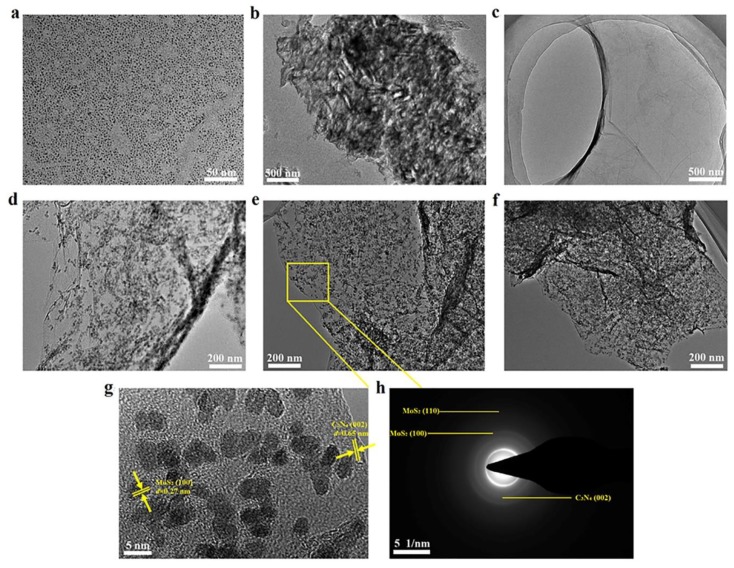
TEM images of MoS_2_ QDs (**a**), bulk g-C_3_N_4_ (**b**), CNNS (**c**), MoS_2_@CNNS(15) (**d**), MoS_2_@CNNS(30) (**e**), and MoS_2_@CNNS(45) (**f**); HRTEM image (**g**) and SAED pattern (**h**) of MoS_2_@CNNS(30).

To obtain more detailed information about the morphologies and microstructures of the as-prepared catalysts, TEM and HRTEM characterizations were carried out. It can be seen in Figure 2a that the MoS_2_ QDs showed a highly homogeneously monodispersed QDs nanostructures with the diameter of about 2–3 nm, indicating the successful preparation of MoS_2_ QDs through the ultrasonic exfoliation method in NMP. Figure 2b presents a typical morphology of slabs with wrinkles of bulk g-C_3_N_4_, and after ultrasonic treatment for 16 h, the three-dimensional (3D) structure of bulk g-C_3_N_4_ was broken up into CNNS with 2D lamellar structure with the thickness of about 2.5 nm (Figure 2c). Thus, the as-prepared 2D CNNS nanosheets exhibited a thinner and explanate lamellar structure, which could provide a larger surface area and more reactive sites in the MoS_2_@CNNS nanocomposites. Figure 2d–f shows the typical TEM images of MoS_2_@CNNS nanocomposites. It can be seen that the MoS_2_ QDs with the diameter of about 2.5–5.5 nm were uniformly distributed on the surface of CNNS nanosheets, and with increasing the amount of Na_2_MoO_4_∙2H_2_O in the ion exchange process, more and larger MoS_2_ QDs were formed. No MoS_2_ QDs were observed except for the surface of CNNS, indicating that the elaborate protonation effect in the ion exchange process leaded to the in-situ nucleation and growth of MoS_2_ QDs on the surface of CNNS. And the large surface area of CNNS provided a nice reaction area, which effectively promoted the in-situ reaction process, resulting in the successful formation of MoS_2_@CNNS nanocomposites. Moreover, the HRTEM image was examined to give further insight into the crystal structure of the MoS_2_@CNNS(30) nanocomposites corresponding to the circled region in Figure 2e. It can be seen in Figure 2g that a lattice spacing of 0.65 nm was obviously shown in the CNNS, which is in accordance with the (002) lattice plane of tetragonal C_3_N_4_. In addition, the QDs displayed well-defined lattice fringes parallel to each other with the same interplanar spacing of 0.27 nm, which can be indexed to the (100) lattice plane of hexagonal-phase MoS_2_. Meanwhile, several apparent diffraction rings were observed in the selected area electron diffraction (SAED) pattern of MoS_2_@CNNS(30) (Figure 2h), which can be well indexed to the lattice planes of MoS_2_ and g-C_3_N_4_, respectively, coinciding well with the XRD results. Therefore, these results indicated that a close-contact and well-defined MoS_2_@CNNS nanostructure was formed via the planar in-situ growth of uniformly-dispersed MoS_2_ QDs on the surface of CNNS during the protonation-assisted ion exchange process, which is a facile and controllable approach to obtain uniform and dispersive MoS_2_ QDs on the surface of substrate.

In order to further probe the contents of S and Mo in MoS_2_@CNNS nanocomposites, ICP-AES was examined. Quantitative analysis results showed the loading amount of MoS_2_ QDs on the surface of CNNS is about 2.0, 5.7 and 5.7 wt % for MoS_2_@CNNS(15), MoS_2_@CNNS(30) and MoS_2_@CNNS(45), respectively (Table S1). These results indicated that with increasing the amount of Na_2_MoO_4_∙2H_2_O in the ion exchange process, the loading capacity and protonated reactive site of CNNS was reaching saturation, which greatly restricted the formation of more MoS_2_ QDs.

In addition, the Brunauer Emmett Teller (BET) specific surface area is a significant affecting factor for the catalytic abilities of catalysts, which was determined by the nitrogen adsorption method [23,24,45]. And the specific surface areas of MoS_2_@CNNS(15), MoS_2_@CNNS(30), MoS_2_@CNNS(45), CNNS, and MoS_2_ QDs were measured as 45.58, 75.93, 69.37, 29.85 and 16.13 m^2^/g, respectively. It can thus be seen that the MoS_2_@CNNS(30) nanocomposites presented a larger specific surface area among the as-prepared catalysts (Figure S1a), though MoS_2_@CNNS(45) loaded a little bit more MoS_2_ QDs, while the agglomeration of the QDs could decrease the specific surface area. As is well known, a larger specific surface area will lead to more active sites, better adsorption performance, and faster electron transfer for catalysts, and will further improve the catalytic activities. Hence, MoS_2_@CNNS(30) nanocomposites are expected to display enhanced peroxidase-like activity.

Furthermore, the zeta potentials of the MoS_2_@CNNS nanocomposites were tested to explore their adsorption properties to molecules with different charges, which is also a significant indicator of the catalytic activities for catalysts. Thus, the zeta potentials of MoS_2_@CNNS nanocomposites were determined as −30.48, −63.38, −53.66, −16.35 and −3.76 mV for MoS_2_@CNNS(15), MoS_2_@CNNS(30), MoS_2_@CNNS(45), CNNS, and MoS_2_ QDs, respectively (Figure S1b). The results indicated that different quantities of MoS_2_ QDs loaded onto the surface of CNNS contributed to different surface charges of MoS_2_@CNNS nanocomposites, which would further lead to different peroxidase-like activities. Therefore, based on the XRD, TEM, ICP-AES, BET, and zeta potential results mentioned above, it can be deduced that the MoS_2_@CNNS nanocomposites prepared via the protonation-assisted ion exchange process showed different morphologies and structures due to the molar ratio of Na_2_MoO_4_∙2H_2_O/CNNS, leading to their different surface/interface properties and further contributing to the distinct peroxidase-like performance.

### 3.2. Peroxidase-Like Activities of MoS_2_@CNNS(30) Nanocomposites

**Figure 3:**
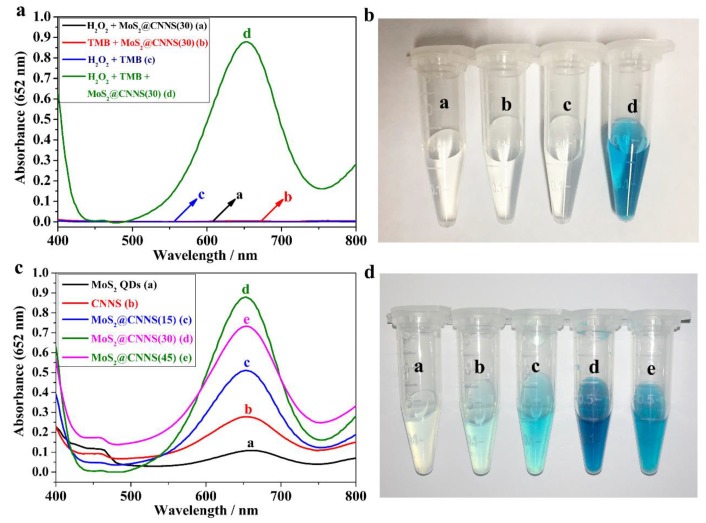
UV-visible absorption spectra (**a**) and color changes (**b**) of different reaction systems (a. H_2_O_2_ + MoS_2_@CNNS(30), b. TMB + MoS_2_@CNNS(30), c. H_2_O_2_ + TMB, and d. H_2_O_2_ + TMB + MoS_2_@CNNS(30); UV-visible absorption spectra (**c**) and color changes (**d**) in the presence of different MoS_2_@CNNS nanocomposites.

To investigate the peroxidase-like activity of MoS_2_@CNNS(30) nanocomposites, the TMB catalytic oxidation experiments were conducted with or without H_2_O_2_ by the UV-visible absorption spectra in the range of 400–800 nm. It can be seen in Figure 3a that low absorption presented in the TMB + MoS_2_@CNNS(30), the H_2_O_2_ + TMB system, and the H_2_O_2_ + MoS_2_@CNNS(30) system, while the H_2_O_2_ + TMB + MoS_2_@CNNS(30) system exhibited an evident absorption peak at 652 nm, indicating that the MoS_2_@CNNS(30) nanocomposites could play a key role in catalyzing the oxidation of TMB in the presence of H_2_O_2_. In addition, the significant color changes of different reaction systems were observed in Figure 3b, which coincided with the absorption spectra. It can be seen that the TMB + MoS_2_@CNNS(30) system, the H_2_O_2_ + TMB system, and the H_2_O_2_ + MoS_2_@CNNS(30) system were almost colorless. However, the H_2_O_2_ + TMB + MoS_2_@CNNS(30) system showed an apparent color variation, presenting a deep blue color, further demonstrating the excellent peroxidase-like properties of the MoS_2_@CNNS(30) nanocomposites.

What’s more, the peroxidase-like activities of other MoS_2_@CNNS, pure MoS_2_ QDs, and CNNS samples were studied and compared via the catalytic oxidation of TMB in the presence of H_2_O_2_. It can be seen in Figure 3c that the MoS_2_@CNNS(30)-based assay system revealed the strongest absorption peak at 652 nm, followed by MoS_2_@CNNS(45), MoS_2_@CNNS(15), CNNS, and MoS_2_ QDs, confirming the best peroxidase-like catalytic activity of MoS_2_@CNNS(30). The color variations of different reaction systems also presented a similar result with the absorption spectra. As shown in Figure 3d, the MoS_2_@CNNS(30)-based assay system showed the deepest blue color compared to that of other materials. Therefore, these results further indicated the enhanced peroxidase-like activities of the MoS_2_@CNNS(30) nanocomposites compared to other MoS_2_@CNNS nanocomposites, pure CNNS, and MoS_2_ QDs. The more negative charge, larger specific surface area, and stable nanostructure of MoS_2_@CNNS(30) mainly facilitated its superior and enhanced peroxidase-like performance, which could increase the binding affinity and absorption to TMB molecules, improve the electron transfer, accelerate the reaction rate, and further improve the peroxidase-like catalytic performance [29]. On the other hand, one can find that both CNNS and MoS_2_ QDs showed a certain intrinsic peroxidase-like catalysis behavior toward TMB-H_2_O_2_ reaction, which could be attributed to the quantum effect and catalysis-active segments of MoS_2_ QDs and CNNS. Thus, when MoS_2_ QDs were loaded on CNNS by the in-situ growth process, the resulting MoS_2_@CNNS(30) nanocomposites could display much higher peroxidase-like activities than pure CNNS and MoS_2_ QDs on account of the synergistic effect of high conductivity and electron transfer capability for CNNS and highly-efficient intrinsic catalytic activity for MoS_2_ QDs [29,36]. The bonding carbon network in CNNS could donate electrons to reduce H_2_O_2_ by accelerating the electron transfer from microcosmic point of view [29]. Furthermore, as a good supporter, CNNS could apparently improve the aqueous dispersion and stability of the nanocomposites. Thus, all of the favorable factors effectively improved the peroxidase-like performances of MoS_2_@CNNS(30) nanocomposites. Hence, these results confirmed the enhanced peroxidase-like activities of the MoS_2_@CNNS(30) nanocomposites, making it a potential highly-efficient colorimetric sensor for H_2_O_2_ detection.

### 3.3. Steady-State Kinetics Assay

**Figure 4:**
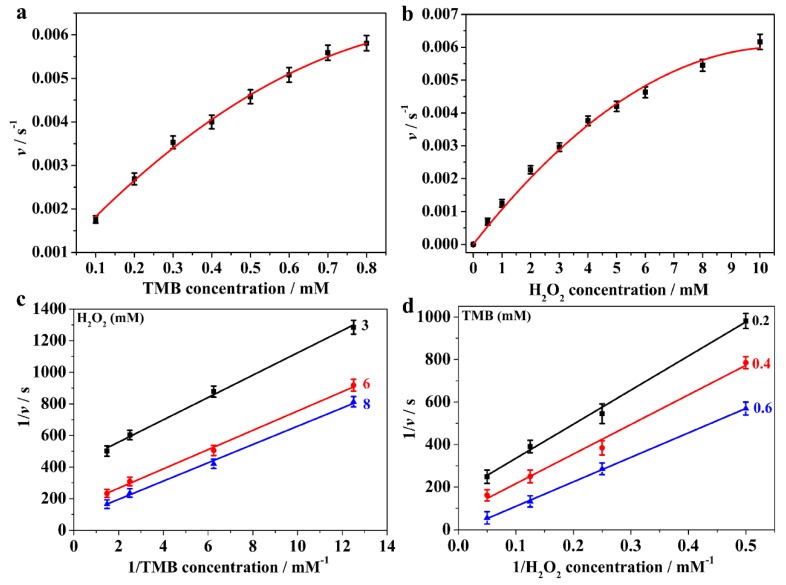
Steady-state kinetic analysis. The reaction velocity (*v*) was measured using 120 μg/mL of MoS_2_@CNNS(30) in 25.0 mM PBS (pH = 4.0) at room temperature. The TMB concentration was varied while the concentration of H_2_O_2_ was 2.0 mM (**a**), the H_2_O_2_ concentration was varied while the concentration of TMB was 0.8 mM (**b**), and the double-reciprocal plots with a fixed concentration of one substrate relative to varying the concentration of the other substrate (**c**) and (**d**).

For further insight into the peroxidase-like catalytic behavior of the MoS_2_@CNNS(30) nanocomposites, the steady-state kinetic assays were carried out with H_2_O_2_ and TMB as substrates. The kinetic data were collected by changing the concentration of one substrate while keeping the other substrate concentration constant [4,42]. Thus, the typical Michaelis-Menten curves were recorded by varying the concentration of TMB or H_2_O_2_ while keeping the other one constant, as shown in Figure 4a,b. The steady-state kinetic reaction parameters were calculated on the basis of the Lineweaver-Burk double reciprocal plot (Figure 4c,d) according to the Michaelis-Menten equation: 1/*v* = (*K*_m_/*V*_max_) × (1/[*S*]) + 1/*V*_max_, where *v* is the initial velocity, K_m_ is the Michaelis-Menten constant, *V*_max_ represents the maximal reaction velocity, and [*S*] signifies the concentration of the substrate [4,29,38,42,46,47], which were listed in Table S2. It is well known that *K*_m_ is an important indicator of the binding affinity of enzyme to the substrates, and that it affects the reaction rate; a smaller value of *K*_m_ generally means a stronger affinity between the enzyme and the substrate [4,29,38,42,46,47]. As listed in Table S2, the *K*_m_ value of MoS_2_@CNNS(30) with TMB was obviously lower than that of the natural enzyme HRP, implying that MoS_2_@CNNS(30) had a stronger binding affinity to TMB than HRP, which might be attributed to the CNNS carriers with strong adsorption to TMB [4,29,38,42]. In addition, the *V*_max_ of MoS_2_@CNNS(30) with TMB was more than two times larger than that of HRP, indicating a favorable tendency of a higher reaction rate, which could the result of the presence of tiny MoS_2_ QDs loaded on the surface of CNNS, as well as the rapid electron transfer capability of CNNS itself [29,36,38]. The *K*_m_ value of MoS_2_@CNNS(30) with H_2_O_2_ was higher than that of HRP, suggesting a lower binding affinity between MoS_2_@CNNS(30) and H_2_O_2_ than that of HRP. Furthermore, the typical Michaelis-Menten behavior towards TMB and H_2_O_2_ with various concentrations in the peroxidase-like catalytic reaction were measured, respectively. And the double-reciprocal plots (Figure 4c,d) of initial velocity against one substrate concentration showed the characteristic parallel lines, indicating a typical ping-pong mechanism of the peroxidase-like catalytic reaction [4,23,24,29,38,42,46,47]. Hence, these results inferred that MoS_2_@CNNS(30) bound and reacted with the first substrate and then released the first product before reacting with the second substrate, which is similar to that of HRP [1,2,3,4,42,48].

### 3.4. Active Species Analysis and Peroxidase-Like Catalytic Mechanism Study

**Figure 5:**
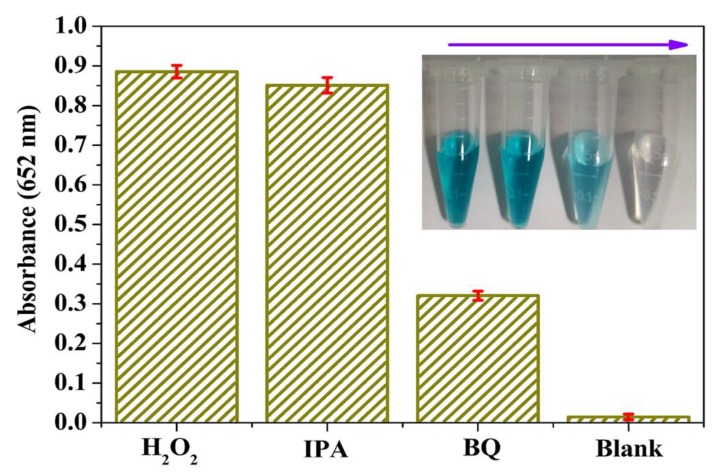
Time-dependent absorbance of reaction solutions at 652 nm in the absence or presence of scavengers (10 mM of IPA and BQ) containing 25.0 mM PBS (pH = 4.0), 2.0 mM H_2_O_2_, 0.8 mM TMB, and 120 μg/mL MoS_2_@CNNS(30) at room temperature. Inset: related color changes.

It is reported that the reaction system contained H_2_O_2_, which would easily produce some active radicals such as ⋅OH and ⋅O_2_^−^ that could play significant roles in catalytic reaction [23,24,49,50]. Hence, in order to explore the peroxidase-like catalytic mechanism of MoS_2_@CNNS(30) nanocomposites, actives species trapping experiments were carried out by adding different scavengers (IPA as ·OH scavengers and BQ as ⋅O_2_^−^ scavengers) into the reaction systems under other fixed experimental conditions. It can be seen in Figure 5 that an evident decrease in absorption and color fading of the reaction system was seen with the addition of BQ, while little change in absorption and color was observed after adding IPA. These results indicated that the MoS_2_@CNNS(30) nanocomposites could catalytically activate H_2_O_2_ to generate ⋅O_2_^−^ radicals in the peroxidase-like catalytic reaction, which subsequently play major roles in oxidizing TMB to produce a TMB oxide with a blue color.

On the basis of the above experimental results and some previous literature [1,2,3,4,23,24,28,29,30,31,32,37,38,39,40], the peroxidase-like catalytic mechanism of MoS_2_@CNNS(30) nanocomposites was proposed, and is illustrated in Scheme 1. It can be seen that the negatively-charged MoS_2_@CNNS(30) nanocomposites could act as the peroxidase mimics, facilitating the electron transfer between TMB and H_2_O_2_ in the catalytic oxidation reaction. During the reaction process, many positively-charged TMB molecules would be absorbed onto the surface of MoS_2_@CNNS(30) nanocomposites owing to the large specific surface area, acting as the chromogenic electron donors. Then, the TMB molecules would donate the lone-pair electrons from the amino groups to the surface of MoS_2_@CNNS(30) nanocomposites, enhancing the density and mobility of electrons on the surface of MoS_2_@CNNS(30), then promoting the electron transfer from MoS_2_@CNNS(30) to H_2_O_2_, and further accelerating the TMB catalytic oxidation reaction rate [4,23,24,28,29,30,31,32,37,38,39,40]. Subsequently, the oxidized intermediate ·O_2_^−^ radicals generated in the reaction between MoS_2_@CNNS(30) and H_2_O_2_ via one electron transfer would react with TMB molecules to generate a TMB oxide, leading to the color change of the system from colorless to blue [4,23,24,28,29,30,31,32,37,38,39,40]. The corresponding chemical equation was: $2H_{2}O_{2}+\;\mathrm{TMB}\;\overset{catalysts}{\to }\;2H_{2}O\;+{\;O}_{2}\;+\;\mathrm{oxTMB}$. Hence, in light of the excellent peroxidase-like catalytic activity, MoS_2_@CNNS(30) nanocomposites exhibited a promising application prospect in medical diagnostics and environmental assay.

### 3.5. Detection of H_2_O_2_ by MoS_2_@CNNS(30)-Based Assay System

**Figure 6:**
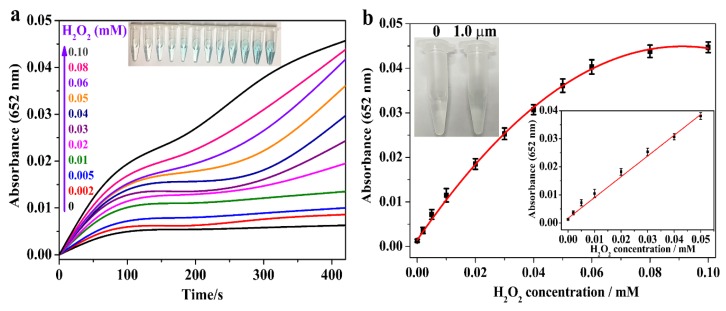
Time-dependent absorbance changes at 652 nm in the presence of different concentrations of H_2_O_2_ in 25.0 mM PBS (pH = 4.0) with 120 μg/mL MoS_2_@CNNS(30) and 0.8 mM TMB at room temperature (**a**) and a dose-response curve for H_2_O_2_ detection (**b**). Inset: related color changes and the linear calibration plot of H_2_O_2_.

On the basis of the aforementioned intrinsic and enhanced peroxidase-like catalytic activity of MoS_2_@CNNS(30), a simple, rapid and ultrasensitive colorimetric method for the visual detection of H_2_O_2_ was developed. As the absorbance of oxidized TMB was in proportion to the H_2_O_2_ concentration, it was a simple approach to determine H_2_O_2_ at 652 nm only by the naked eye, or by using an UV-visible spectrometer. Figure 6a shows the time-course dependent absorbance changes at 652 nm of the oxidized TMB in the presence of H_2_O_2_ with different concentrations. It can be seen that the reaction rate increased by increasing the concentration of H_2_O_2_ from 0.002 mM to 0.10 mM. Moreover, it can be seen in Figure 6b that the typical H_2_O_2_ concentration-response curve displayed a linear response in the range of 2.0 μM to 50.0 μM, and the linear fitting equation is$\;A_{652\mathrm{nm}}\;=\;1.63\times 10^{-3}\;+\;0.748C$ (mM), with a correlation coefficient of 0.9956. Hence, the detection limit of H_2_O_2_ was estimated to be 0.02 μM. In addition, as shown in the inset of Figure 6b, the color variation for H_2_O_2_ response as low as 1.0 μM was also apparently observed by the naked eye, which offered a convenient approach to detect H_2_O_2_ even at low concentrations. Furthermore, compared to some other previously-reported nanomaterials with peroxidase-like activities [17,25,29,30,37,39,42,46,51] (Table S3), MoS_2_@CNNS(30) nanocomposites revealed a reasonable linear range and a lower detection limit for H_2_O_2_ detection, further confirming their superior peroxidase-like catalytic activity and sensitivity. Therefore, these results indicated that the visual biosensing platform based on MoS_2_@CNNS(30) was a simple, rapid, and convenient method for H_2_O_2_ detection with ultrasensitive response.

### 3.6. Selectivity and Applicability of MoS_2_@CNNS(30)-Based Assay System

**Figure 7:**
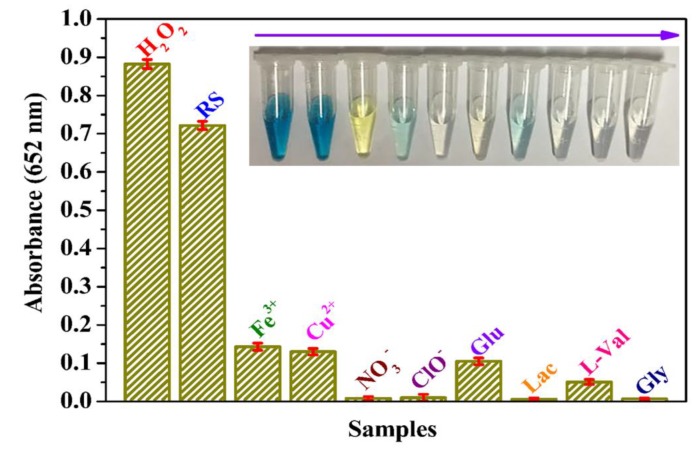
Reveal sample (antiseptic liquid was diluted 1000 times) and selectivity analysis with interfering substances (20.0 mM of Fe^3+^, Cu^2+^, NO_3_^−^, ClO^−^, Glu, Lac, L-Val, and Gly) of MoS_2_@CNNS(30)-based assay system by monitoring the absorbance at 652 nm. Inset: related color changes.

To estimate the selectivity of MoS_2_@CNNS(30)-TMB-H_2_O_2_ detection system, some other substances, such as Fe^3+^, Cu^2+^, NO_3_^−^, ClO^−^, Glu, Lac, L-Val, and Gly, with the concentration of 20.0 mM were added into the reaction system respectively as the potential interferents instead of H_2_O_2_. In addition, a commercial antiseptic liquid (diluted 1000 times) was used to check the practical applicability and accuracy of the H_2_O_2_ detection system in a real sample (RS). It can be seen in Figure 7 that no evident absorbance at 652 nm and color change were observed, though the concentrations of these interferents were 10 times higher than that of standard H_2_O_2_ (2.0 mM), indicating a nice selectivity of the detection system. Moreover, the diluted commercial antiseptic liquid presented an obvious absorbance and light blue color, and the concentration of H_2_O_2_ in the commercial antiseptic liquid could be calculated to be about 0.77 M based on the calibration curve shown in Figure 6b, which was close to the actual concentration of the commercial antiseptic liquid (0.79 M). Therefore, these results exhibited the favorable applicability in complicated conditions of the MoS_2_@CNNS(30)-based H_2_O_2_ detection system, which favored the practical and rapid determination of H_2_O_2_ in various environments.

### 3.7. Stability and Reusability of MoS_2_@CNNS(30)-Based Assay System

**Figure 8:**
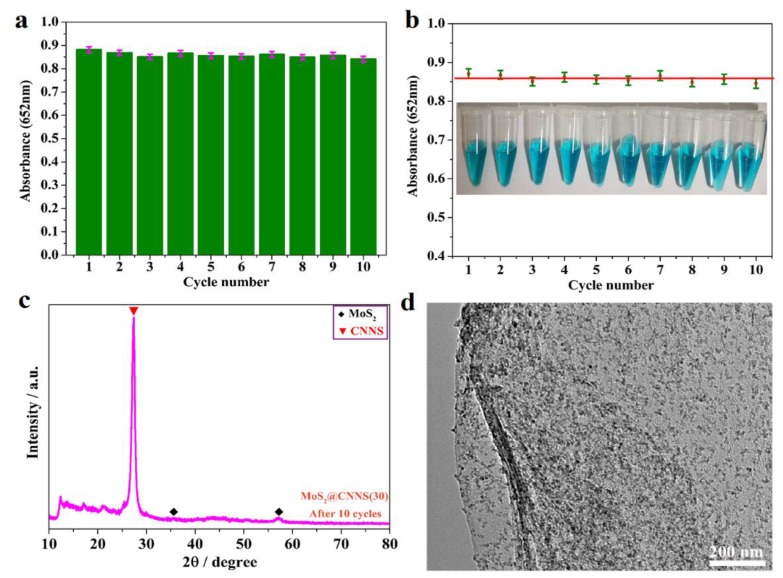
Stability and reusability experiments of MoS_2_@CNNS(30)-based assay system containing 120 μg/mL MoS_2_@CNNS(30), 25.0 mM PBS (pH = 4.0), 2.0 mM H_2_O_2_, and 0.8 mM TMB (**a**), (**b**); XRD pattern (**c**) and TEM image (**d**) of MoS_2_@CNNS(30) after 10 cycles. Inset: related color changes in 10 cycles.

In order to study the stability and reusability of MoS_2_@CNNS(30)-based H_2_O_2_ detection system, the peroxidase-like experiments were conducted by repeating the reaction for ten successive cycles. After each cycle, the MoS_2_@CNNS(30) samples were collected, washed several times with Milli-Q water and ethanol, and dried, and then reused in the next cycle. Every cycle lasted for 10 min. It can be seen in Figure 8a,b that there was no significant change of the reaction system absorbance at 652 nm during the recycling tests, accompanied by no color variations of the reaction system observed in the recycling tests, showing the excellent reusability and stable peroxidase-like performance of the MoS_2_@CNNS(30)-based H_2_O_2_ detection system. The relative standard deviation (RSD) of the absorbance values was only 1.72%, which further confirmed the nice reproducibility, stability, and reusability of the MoS_2_@CNNS(30)-based assay system even for 10 cycles. Moreover, XRD and TEM were used to further analyze the crystal structure and morphology of MoS_2_@CNNS(30) after ten successive cycles. It can be seen in Figure 8c that the XRD pattern of MoS_2_@CNNS(30) after ten successive cycles displayed no apparent change in both peak intensity and position, implying the stable crystal structure of MoS_2_@CNNS(30) samples kept in the recycling tests. In addition, no significant morphology variation was observed in the TEM image of MoS_2_@CNNS(30) samples after ten successive cycles (Figure 8d), though a little impurity appeared on the surface of CNNS, exhibiting the good stability in crystal structure and morphology. Hence, good stability, reusability, reproducibility and precision of the MoS_2_@CNNS(30)-based assay system suggested that the peroxidase-like colorimetric method might be used to analyze H_2_O_2_ in real water samples, which also favored long-term use.

## 4. Conclusions

In conclusion, the MoS_2_@CNNS nanocomposites were successfully synthesized via a protonation-assisted ion exchange method, which were demonstrated to display an enhanced intrinsic peroxidase-like activity. The in-situ growth of MoS_2_ QDs on the surface of ultrathin CNNS formed a stable nanostructure, facilitating the synergetic effects of high conductivity and electron-transfer capability for CNNS and intrinsic catalytic activity for MoS_2_ QDs, and thus, greatly improving the peroxidase-like performance of the nanocomposites. In addition, the MoS_2_@CNNS nanocomposites revealed different morphologies, surface properties, and peroxidase-like activities, owing to the different amount of MoS_2_ QDs loading on the surface of CNNS. The MoS_2_@CNNS(30) nanocomposites showed the best peroxidase-like ability among the composites, which could be attributed to their more negative potential and larger specific surface area. In the presence of H_2_O_2_ and the peroxidase substrate TMB, MoS_2_@CNNS(30) could induce a typical blue color reaction, thus providing a colorimetric assay for H_2_O_2_. The catalytic activity was strongly dependent on the catalyst concentration, H_2_O_2_ concentration, pH, and temperature. Moreover, the kinetic and active species trapping experiments indicated that the catalytic reaction followed a ping-pong mechanism, and the ⋅O_2_^−^ radicals played a pivotal role in the peroxidase-like catalytic reaction. Based on the excellent peroxidase-like catalytic performance of MoS_2_@CNNS(30) nanocomposites, a simple, rapid, and ultrasensitive platform for colorimetric detection of H_2_O_2_ was developed, which exhibited a nice selectivity, reusability, long-term stability, and practicability, making it a potential biosensing material for the practical applications in H_2_O_2_ detection and biomedical analysis. Furthermore, this work offers some new insights and targeted directions for novel enzymatic mimics with enhanced catalytic activity.

## Supplementary Materials

The following are available online at http://www.mdpi.com/2079-4991/8/12/976/s1, Figure S1: Adsorption/desorption isotherms of MoS_2_@CNNS(30) (a) and zeta potentials of the MoS_2_@CNNS nanocomposites dispersed in ultrapure water (pH = 4.0) (b), Figure S2: Time-dependent absorbance at 652 nm and color changes of 0.8 mM TMB reaction solutions in the absence or presence of different concentrations of MoS_2_@CNNS(30) (a) and H_2_O_2_ (b) in 25.0 mM PBS (pH = 4.0) at room temperature. Inset: related color variations, Figure S3: Dependency of peroxidase-like activity of MoS_2_@CNNS(30) on pH (a) and temperature (b) and color changes. Experiments were conducted by using 120 μg/mL of MoS_2_@CNNS(30) in 25.0 mM PBS with 2.0 mM H_2_O_2_ and 0.8 mM TMB as substrates; Inset: related color variations, Table S1: MoS_2_ loading amount in MoS_2_/CNNS samples determined by ICP-AES, Table S2: Comparison of *K*_m_ and *V*_max_ between MoS_2_@CNNS(30) and HRP for H_2_O_2_ and TMB, Table S3: Comparison of peroxidase-like activity in the linear range and detection limit of H_2_O_2_ between MoS_2_@CNNS(30) and other peroxidase mimics.
