# Supplementary material for: Enhanced Peroxidase-Like Activity of MoS2 Quantum Dots Functionalized g-C3N4 Nanosheets towards Colorimetric Detection of H2O2
Source: Nanomaterials (Basel). 2018 Nov 26;8(12):976. doi: 10.3390/nano8120976 (PMC6316126; doi:10.3390/nano8120976)
Supplement: Supplementary file 1 [file nanomaterials-08-00976-s001.pdf]

nanomaterials

Supplementary Information

Enhanced Peroxidase-Like Activity of MoS_2 Quantum Dots Functionalized g-C_3N_4 Nanosheets towards Colorimetric Detection of H_2O_2

1. BET and zeta potential analysis

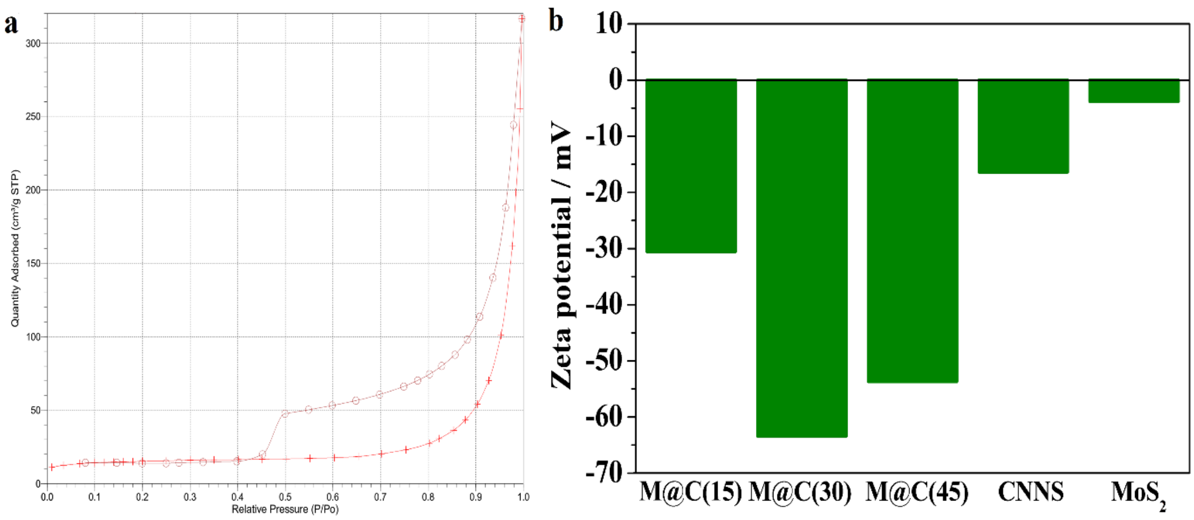

Figure S1. Adsorption/desorption isotherms of MoS_2@CNNS(30) (a) and zeta potentials of the MoS_2@CNNS nanocomposites dispersed in ultrapure water (pH = 4.0) (b).

2. Optimization of experimental conditions

The effect of MoS_2@CNNS(30) concentration (0~200 μg/mL), H_2O_2 concentration (0~5.0 mM), temperature (10~50 °C), and pH (2.0~9.0) on the peroxidase-like activity of MoS_2@CNNS(30) were assayed with the same procedures to the peroxidase mimetic experiments to obtain the optimal reaction conditions. Typically, the tests were performed by in sequence adding 500 μL of 50.0 mM phosphate buffer solution (PBS, pH = 2.0~9.0), 100 μL of 8.0 mM TMB, 200 μL H_2O_2 with the final concentration of 0~5.0 mM, and 200 μL MoS_2@CNNS(30) dispersion with the final concentration of 0~200 μg/mL under the temperature range of 10~50 °C. Make three variable fixed and change another one to obtain the optimal experimental conditions. Then the reaction systems were monitored in a time-scan mode at 652 nm by an UV-visible spectrophotometer (Shimadzu UV-2500, Japan) right after all of the components were added and mixed.

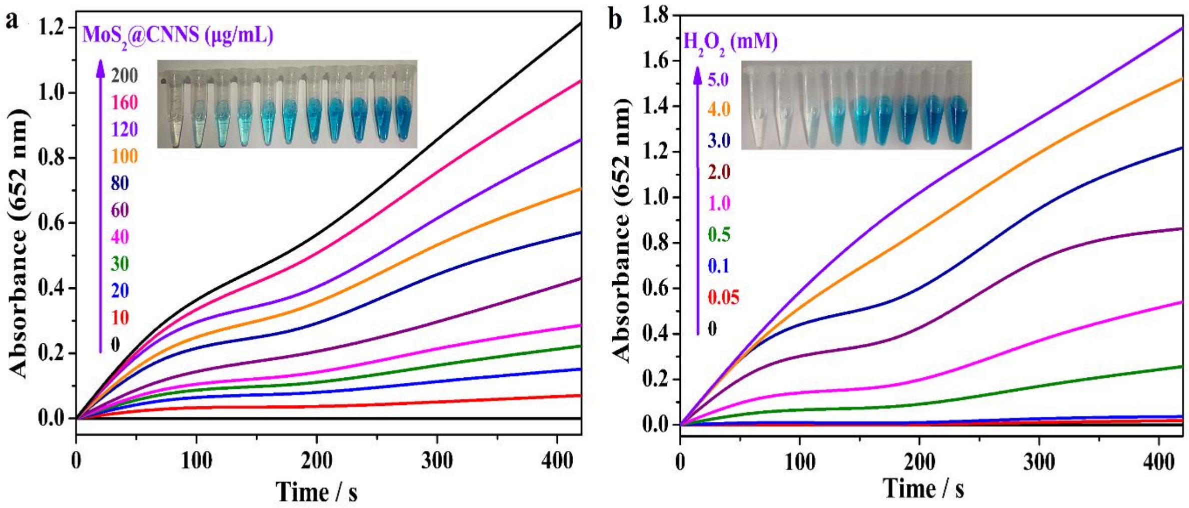

Figure S2. Time-dependent absorbance at 652 nm and color changes of 0.8 mM TMB reaction solutions in the absence or presence of different concentrations of MoS_2@CNNS(30) (a) and H_2O_2 (b) in 25.0 mM PBS (pH = 4.0) at room temperature. Inset: related color variations.

Figure S2 shows the catalytic activity tests under different catalyst concentrations and H_2O_2 concentrations. It can be seen that the catalytic reaction rate obviously increased as increasing the concentration of MoS_2@CNNS(30) catalysts with an apparent color variation (**Figure S2(a)**). Hence, in view of the operation convenience, the concentration of 120 μg/mL for MoS_2@CNNS(30) was selected as the optimal concentration. The effects of H_2O_2 concentration on the catalytic activity of MoS_2@CNNS(30) were also tested (**Figure S2(b)**). It can be seen that the catalytic reaction rate increased with the increase of H_2O_2 concentration, and there was no inhibition in the catalytic reaction at high H_2O_2 concentration, indicating a more stable enzyme catalytic activity of MoS_2@CNNS(30) than that of horseradish peroxidase (HRP) at high H_2O_2 concentration [S1]. Therefore, the H_2O_2 concentration of 2.0 mM was chosen with a medium and visual absorbance. In addition, **Figure S2(b)** shows the color changes with different H_2O_2 concentration in the reaction system, illustrating that with the increase of H_2O_2 concentration the color changed from light to dark blue, which further demonstrated the feasibility of H_2O_2 detection through a colorimetric method.

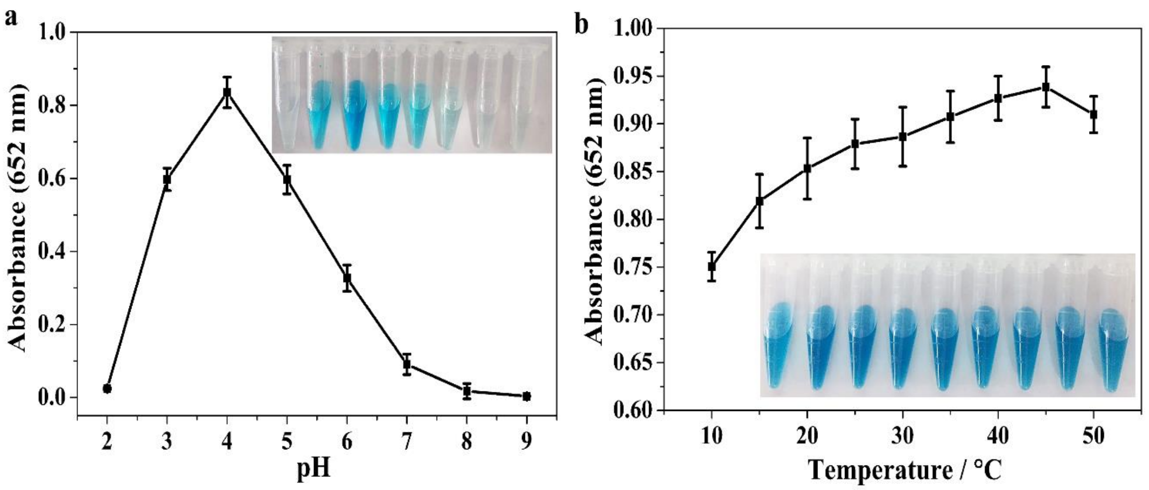

Figure S3. Dependency of peroxidase-like activity of MoS_2@CNNS(30) on pH (a) and temperature (b) and color changes. Experiments were conducted by using 120 μg/mL of MoS_2@CNNS(30) in 25.0 mM PBS with 2.0 mM H_2O_2 and 0.8 mM TMB as substrates. Inset: related color variations.

Furthermore, similar to natural enzymes, the catalytic activity of MoS_2@CNNS(30) is found to be pH-dependent, which was tested by UV-visible spectrophotometer with changing the pH values from pH 2.0 to 9.0. It can be seen in **Figure S3(a)** that the optimal pH value of the reaction was found to be pH 4.0, while the pH value above or lower than 4.0 would lower the peroxidase-like activity. Thus, the optimal pH was 4.0. Moreover, **Figure S3(b)** shows a temperature-dependent assay in the range of 10 °C to 50 °C. It can be seen notably that the reaction system was affected by the temperature, and the catalytic activity exhibited a continue rising tendency, indicating that the inorganic enzyme mimics could present high thermal activities. Therefore, considering the convenience of operation, the room temperature 25 °C was selected as the experimental temperature. The results also indicated the relatively stable peroxidase-like activity of MoS_2@CNNS(30) even under fairly harsh conditions.

Table S1. MoS_2 loading amount in MoS_2/CNNS samples determined by ICP-AES.

Sample	Composite (g/L)	Mo (mg/L)	S (mg/L)	MoS_2/composite (wt%)
MoS_2/CNNS(15)	1.0	12.2	8.2	2.0
MoS_2/CNNS(30)	1.0	34.7	22.3	5.7
MoS_2/CNNS(45)	1.0	34.8	22.4	5.7

Table S2. Comparison of Km and Vmax between MoS_2@CNNS(30) and HRP for H_2O_2 and TMB.

Catalyst	Substance	K_m (mM)	V_{max} (M/s)	Reference
MoS_2@CNNS(30)	H_2O_2	0.602	3.15×10^{-8}	This work
MoS_2@CNNS(30)	TMB	0.117	3.03×10^{-8}	This work
HRP	H_2O_2	0.214	2.46×10^{-8}	S2, S3
HRP	TMB	0.275	1.24×10^{-8}	S2, S3

Table S3. Comparison of peroxidase-like activity in the linear range and detection limit of H_2O_2 between MoS_2@CNNS(30) and other peroxidase mimics.

Mimetic enzyme	Linear range (μM)	Detection limit (μM)	Reference
MoS_2@CNNS(30)	2~50	0.02	This work
Fe_3O_4	5~100	3.0	S4
Co-Al LDH	10~50	10.0	S5
WS_2 Nanosheets	5~200	1.5	S6
g-C_3N_4	5~100	1.0	S7
MoS_2 Nanoparticles	2~100	0.32	S8
MoS_2 Nanoflakes	0.125~1.75	4.1	S9
MoS_2@$MgFe_2O_4$	2.5~300	1.0	S10
Fe-g-C_3N_4	0.5~10	0.05	S11
MnSe-g-C_3N_4	18~1800	1.8	S12

References

S1. Nicell, J.A.; Wright, H. A model of peroxidase activity with inhibition by hydrogen peroxide. *Enzyme Microb. Tech.* **1997**, *21*, 302-310, DOI: 10.1016/S0141-0229(97)00001-X.

S2. Lin, Y.; Ren, J.; Qu, X. Catalytically active nanomaterials: a promising candidate for artificial enzymes. *Acc. Chem. Res.* **2014**, *47*, 1097-1105, DOI: 10.1021/ar400250z.

S3. Qiao, F.M.; Chen, L.J.; Li, X.; Li L.; Ai, S.Y. Peroxidase-like activity of manganese selenide nanoparticles and its analytical application for visual detection of hydrogen peroxide and glucose. *Sensor. Actuator. B: Chem.* **2014**, *193*, 255-262, DOI: 10.1016/j.snb.2013.11.108.

S4. Wei, H.; Wang, E.K. Fe_3O_4 magnetic nanoparticles as peroxidase mimetics and their applications in H_2O_2 and glucose detection. *Anal. Chem.* **2008**, *80*, 2250-2254, DOI: 10.1021/ac702203f.

S5. Chen, L.J.; Sun, B.; Wang, X.; Qiao, F.M.; Ai, S.Y. 2D ultrathin nanosheets of Co-Al layered double hydroxides prepared in L-asparagine solution enhanced peroxidase-like activity and colorimetric detection of glucose. *J. Mater. Chem. B* **2013**, *1*, 2268-2274, DOI: 10.1039/C3TB00044C.

S6. Chen, Q.; Chen, J.; Gao, C.J.; Zhang, M.L.; Chen J.Y.; Qiu, H.D. Hemin-functionalized WS_2 nanosheets as highly active peroxidase mimetics for label-free colorimetric detection of H_2O_2 and glucose. *Analyst* **2015**, *140*, 2857-2863, DOI: 10.1039/c5an00031A.

S7. Lin, T.R.; Zhong, L.S.; Wang, J.; Guo, L.Q.; Wu, H.Y.; Guo, Q.Q.; Fu, F.F.; Chen, G.N. Graphite-like carbon nitrides as peroxidase mimetics and their applications to glucose detection. *Biosens. Bioelectron.* **2014**, *59*, 89-93, DOI: 10.1016/j.bios.2014.03.023.

S8. Zhao, K.; Gu, W.; Zheng, S.S.; Zhang, C.L.; Xian, Y.Z. SDS-MoS_2 nanoparticles as highly-efficient peroxidase mimetics for colorimetric detection of H_2O_2 and glucose. *Talanta* **2015**, *141*, 47-52, DOI: 10.1016/j.talanta.2015.03.055.

S9. Yu, J.; Ma, D.Q.; Mei, L.Q.; Gao, Q.; Yin, W.Y.; Zhang, X.; Yan, L.; Gu, Z.J.; Ma, X.Y.; Zhao, Y.L. Peroxidase-like activity of MoS_2 nanoflakes with different modifications and their application for H_2O_2 and glucose detection. *J. Mater. Chem. B* **2018**, *6*, 487-498, DOI: 10.1039/C7TB02676E.

S10. Zhang, Y.; Zhou, Z.F.; Wen, F.F.; Tan, J.; Peng, T.; Luo, B.Q.; Wang, H.G.; Yin, S.X. A flower-like MoS_2-decorated $MgFe_2O_4$ nanocomposite: Mimicking peroxidase and colorimetric detection of H_2O_2 and glucose. *Sensor. Actuator. B: Chem.* **2018**, *275*, 155-162, DOI: 10.1016/j.snb.2018.08.051.

S11. Tian, J.Q.; Liu, Q.; Asiri, A.M.; Qusti, A.H.; Al-Youbi, A.O.; Sun, X.P. Ultrathin graphitic carbon nitride nanosheets: a novel peroxidase mimetic, Fe doping-mediated catalytic performance enhancement and application to rapid, highly sensitive optical detection of glucose. *Nanoscale* **2013**, *5*, 11604-11609, DOI: 10.1039/C3NR03693F.

S12. Qiao, F.M.; Qi, Q.Q.; Wang, Z.Z.; Xu, K.; Ai, S.Y. MnSe-loaded g-C_3N_4 nanocomposite with synergistic peroxidase-like catalysis: Synthesis and application toward colorimetric biosensing of H_2O_2 and glucose. *Sensor. Actuator. B: Chem.* **2016**, *229*, 379-386, DOI: 10.1016/j.snb.2015.12.109.
